# PCB153-Induced Overexpression of ID3 Contributes to the Development of Microvascular Lesions

**DOI:** 10.1371/journal.pone.0104159

**Published:** 2014-08-04

**Authors:** Jayanta K. Das, Quentin Felty

**Affiliations:** Department of Environmental & Occupational Health, Florida International University, Miami, Florida, United States of America; University of Kentucky Medical Center, United States of America

## Abstract

Microvascular lesions resulting from endothelial cell dysfunction are produced in the brain, lung, kidney, and retina of patients of complex chronic diseases. The environmental and molecular risk factors which may contribute in the development of microvascular damage are unclear. The mechanism(s) responsible for initiating microvascular damage remain poorly defined, although several inciting factors have been proposed, including environmental toxicants-induced oxidative stress. Enhanced neovascularization has been implicated in either the development or progression of proliferative vascular lesions. Here, we present evidence for how PCB-induced ROS may contribute to the development of a neovascular phenotype with the aim of elucidating the role of environmental toxicants in endothelial dysfunction with a specific focus on the inhibitor of differentiation protein ID3. We used a combination of phenotype and immunohistochemical analysis followed by validating with protein expression and post-translational modifications with Western Blot and MALDI-TOF/TOF analysis. We also looked for a correlation between ID3 expression in vascular tissue. Our results showed that PCB-induced ROS mediated a highly tube branched neovascular phenotype that also depended on ID3 and Pyk2; and PCB153 treatment increased the size of endothelial spheroids under conditions typically used for clonal selection of stem cell spheroids. High ID3 protein expression correlated with a greater degree of malignancy and oxidative DNA damage marker 8-OHdG in blood vessels from human subjects. PCB153 treatment increased both serine and tyrosine phosphorylation of endothelial ID3. Stable ID3 overexpression increased cell survival of human microvascular endothelial cell line hCMEC/D3. In summary, our data provide evidence that ID3 may play a critical role in regulating vascular endothelial cell survival and development of microvascular lesions induced by persistent environmental pollutants such as PCB153. Findings of this study are important because they provide a new paradigm by which PCBs may contribute to the growth of microvascular lesions.

## Introduction

The endothelium is a highly metabolically active organ weighing approximately 1 kg and covering a total surface area of 4000–7000 m^2^ in an average-sized human [1]. It is noteworthy that the underexplored microvasculature represents the largest fraction of the total vascular cross-sectional area and may hold important clues that link environmental pollutants to vascular disease. Microvascular complications have been shown to contribute to the development of cardiovascular disease, diabetic retinopathy, and neurological disease. Furthermore, proliferative microvascular lesions that result from a focal budding of endothelial cells (ECs) have been reported to be an aggressive endothelial phenotype associated with a poor prognosis in glioblastoma multiforme, non-small cell lung cancer, and severe idiopathic pulmonary arterial hypertension [2]–[4]. The risk factor and possible molecular mechanisms responsible for the development of microvascular lesions are still largely unknown. Since environmental polychlorinated biphenyls (PCBs) have been reported in human blood [5]; vascular toxicity from PCB exposure is a public health concern. Evidence in support of vascular toxicity from PCBs comes from epidemiological studies that have shown the link between PCB exposure and risk for vascular disease [6]–[9]. However, how exposure to PCBs may affect the development of microvascular lesions is not clear. PCBs are a class of commercially synthesized chemicals that were used worldwide for electrical equipment, hydraulic machinery, and as additives in pesticides, paints, ink, and plastics during the 1930s through the mid-1970s. The stability of these chemicals has allowed them to persist in the environment and consequently continues to expose the general population to PCBs mainly via the food-chain. In the present study, we determined whether PCB-induced reactive oxygen species (ROS) contributed to the development of an endothelial-like lesion.

We have previously shown that ID3 is an important determinant of ROS-induced proliferation of estrogen exposed ECs [10], however, the effect of environmental PCBs on Id proteins has not been investigated until now. The Id (inhibitors of DNA binding and differentiation) family of proteins family of proteins consists of four genes (Id1–Id4). ID3 is expressed in ECs during embryogenesis, but is down-regulated as tissues mature [11]. Id protein-protein interactions occur via the helix-loop-helix (HLH) motif in which Id proteins dimerize with basic HLH transcription factors. This type of Id interaction has been reported to regulate transcription in a dominant-negative manner [12]. Id proteins have also been reported to bind to proteins that do not contain the HLH motif such as caveolin-1 [13]. Besides negative regulation of cell differentiation, Id proteins also act as positive regulators of cell growth and play a major role in neovascularization [12]. Since ID3 is a redox-sensitive gene that has been implicated in vascular lesion formation [14]–[16], we hypothesize that ID3 is involved in the development of the microvascular lesion in response to the environmental toxicants. Here, we have elucidated the role of environmental PCBs in endothelial dysfunction with a specific focus on ID3 by showing evidence that ID3 may play a critical role in regulating vascular endothelial cell survival and development of microvascular lesions induced by the persistent environmental pollutant, PCB153. A better understanding of how microvascular lesions depend on ID3 may open up new avenues for prevention and treatment of diseases that harbor microvascular lesions.

## Materials and Methods

### Cell culture and treatment conditions

Primary cells, Human umbilical vein endothelial cells (HUVECs) and normal human dermal fibroblasts (NHDF), were purchased from Cambrex. HUVECs (passages 2–10) were maintained in endothelial cell basal medium-2 (EBM-2) supplemented with EGM2-MV SingleQuots (Cambrex). NHDF (passages 3–6) were maintained in monoculture in EBM-2 supplemented with FGM-2 SingleQuots (Cambrex). The effect of PCB treatment on HUVECs was studied under the following culture conditions: HUVECs were starved in phenol red-free mammary epithelium basal medium in the absence of serum and growth factors for 3 h. Thereafter, the cells were treated with PCB153 (2,2′,4,4′,5,5′-hexachlorobiphenyl) and PCB77 (3,3′,4,4′-tetrachlorobiphenyl) for the indicated time periods. PCB congeners were purchased from AccuStandard (New Haven, CT) and dissolved in dimethyl sulfoxide (DMSO). Equal volumes of DMSO as in PCB treatment group were added to vehicle control with the final percentage of DMSO in each group to be less than 0.1%. The effect of ROS scavengers (N-acetylcysteine and ebselen) was studied by pretreating the cells for 2 h prior to PCB exposure. For ROS scavenger enzyme studies, HUVECs were incubated with replication-deficient adenoviral constructs containing human MnSOD and catalase (Ad-MnSOD and Ad-CAT, ViraQuest Inc) at 50 multiplicities of infection (MOI) for 24 h. The immortalized human cerebral microvascular endothelial cell line hCMEC/D3 was obtained from Dr. B. Weksler, Weill Medical College of Cornell University, NY. The hCMEC/D3 cells have been shown to retain their EC characteristics with a stable normal karyotype, proliferate in response to endothelial growth factors, and form capillary tubes in matrix but no colonies in soft agar [17]. The hCMEC/D3 cell line was maintained in DMEM-F12 media with 5% FBS. Cells were cultured at 37°C in a humidified atmosphere with 5% CO_2_.

### Endothelial tube assay on matrix gel

HUVECs were seeded on top of the solidified Cultrex Matrix (Trevigen) in the presence or absence of E2 for 5 h. Tube formation on the Cultrex Matrix was observed under the microscope and photographed with an Olympus C-5060 digital camera. AdobePhotoshop was used for conversion to black and white photos. Triplicates were used in all experiments and each experiment was repeated at least three times. For quantification of tube formation the number of branch points of the formed tubes was counted as described by Soares et al. [18]. Data shown are expressed as mean ± SD and statistical significance was determined using Student's *t*-test with *P*-values smaller than 0.05 considered as significant.

### Co-culture tube assay

In a 24-well plate, HUVECs were seeded on top of NHDF in phenol red-free mammary epithelium basal medium. Simultaneously, cells were treated with PCBs, ROS scavengers, or the vehicle control (0.1% DMSO) for 3 days. The ELISA was performed as described by Friis et al. [19] The endothelial tube structures were photographed and quantified as described previously. Triplicates were used in all experiments and each experiment was repeated at least three times. For RNA interference (RNAi) studies, HUVECs were cultured in 6-well plates to 60% confluency. Transfection was performed using FuGENE 6 (Roche) according to the manufacturer's protocol. ID3 siRNA and Pyk2 siRNA was designed, synthesized, and annealed by Ambion with the following sequences: ID3 Sense: 5′-GGAGCUUUUGCCACUGACUTT-3′, ID3 Antisense: 5′-AGUCAGUGGCAAAAGCUCCTT-3′; and Pyk2 Sense: 5′-GGUCUGCUUCUAUAGCAACTT-3′, Pyk2 Antisense: 5′-GUUGCUAUAGAAGCAGACCTT-3′. The FuGENE:siRNA mixture (100 µl/well) was added to HUVECs in EBM-2 and returned to the incubator for 48 h prior to co-culture experiments. Silencer negative control siRNA (Ambion) was used in all RNAi experiments.

### Endothelial spheroid assay

Cells were suspended in serum-free DMEM/F12 (1∶1) culture medium supplemented with B27. For EC spheroid formation, approximately 100–150 cells per well were seeded in an ultra low-attachment 96-well plate (Corning Inc, Lowell, MA). The effect of ROS scavenger N-acetylcysteine was determined by pre-treating the cells with 3 mM NAC 1 h prior to exposure with 60 µM PCB153 on the day of seeding cells. Endothelial spheroids were grown for 10 days in liquid culture in the absence or presence of PCB153. A total of 15 endothelial spheroids with a minimum diameter of 50 µm were counted in each experimental group. Data were analyzed by ANOVA; Tukey HSD test for multiple comparisons.

### Tissue microarray analysis of ID3 expression

Immunohistochemistry: ID3 expression was determined in a cardiovascular disease tissue microarray designed for blood vessel or endothelial cell related studies (Cat. #CVD481, US Biomax, Inc). For immunohistochemical preparation: In brief, paraffin embedded tissue sections were placed in xylene and were gradually re-hydrated in successive steps with ethanol (100%, 95%, 70%) and PBS, pH 7.4. Then tissues were blocked with 3% normal goat serum and incubated with antibodies: anti-ID3 overnight at 4°C. After washing with 1X PBS, tissues were incubated with anti-rabbit-IgG were counterstained with hematoxylin QS and with serially dehydrated in ethanol (25%, 50%, 75%, 95% & 100%). Then tissues were dipped in xylene and placed on cover slip with histological mounting medium.

Immunofluorescence preparation: Paraffin embedded tissue sections were placed in xylene and were gradually re-hydrated in successive steps with ethanol (100%, 95%, 70%), PBS, pH 7.4. and treated for antigen unmasking with 10 mM sodium citrate buffer, pH 6.0. Then slides were incubated in 3% normal goat serum and washed in PBS with 0.5% Tween 20 (PBST). Slides were incubated with primary antibodies anti-ID3 and anti-8-hydroxydeoxyguanosine (8-OHdG) for overnight at 4°C, washed in PBST and incubated with anti-rabbit-IgG Alexa Fluor 546 for ID3 and anti-mouse-IgG Alexa Fluor 633 for 8-OHdG, washed in PBST and mounted with Fluoromount-G slide mounting medium. Images were captured with DeltaVision ELITE Olympus IX71 fluorescence microscope, Applied precision (Thermo Scientific) using software softWoRx-Acquire Version: 5.5.1 Release 3. Immunostaining of ID3 was semi-quantitatively evaluated by assigning a score for the intensity of the immunohistochemical reaction and for the proportion of the ID3 cells stained as previously described by Wilson *et al.* The product of these two values was taken to give the overall IR (total score). The intensity of the immunohistochemical reaction (intensity score) was stratified into four categories: 0, no IR; 1, weak IR; 2, moderate IR; and 3, strong IR. All statistics were performed using Spearman Rank Correlation (v1.0.1) in free Statistics Software (v1.1.23-r7), Office for Research Development and Education, by Wessa P (2012), website: http://www.wessa.net/rwasp_spearman.wasp accessed on 02/18/2014.

### Immunoblotting

Whole cell lysates were prepared with lysis buffer containing [25 mM Tris-HCl buffer (pH 8.0), 150 mM NaCl, 0.2% NP-40, 10% glycerol, 8 mM β-glycerophosphate, 2.5 mM sodium pyrophosphate, 10 mM NaF, 0.2 mM Na_3_VO_4_, 1 mM DTT and 10 µl/ml protease inhibitor cocktail (Sigma Aldrich). Proteins were quantified using the Bradford Assay Reagent (Bio-Rad) according to the manufacturer's instructions. Proteins (35–75 µg) were separated by 10% SDS-PAGE and transferred to polyvinylidene fluoride (PVDF) membranes (Millipore). Membranes were blocked with 5% nonfat milk and incubated with the following antibodies: ID3 (Cal BioReagent), CD31, pY^402^-Pyk2, Pyk2, and p-Ser (Santa Cruz BioTechnology); p-Tyr and β-actin (Cell Signaling). Antibody dilutions used were according to manufacturer's recommendations for detection by immunoblot. Membranes were then incubated with horseradish peroxidase-conjugated secondary IgG antibodies and visualized with ECL Plus Western blot reagents (GE Healthcare, Amersham). The membranes were re-probed for β-actin as loading control. Electrochemiluminescence (ECL) intensity of detected target proteins was imaged and quantified with a Bio-Rad Versa Doc instrument. All immunoblots were completed a minimum of three times for each experiment.

### Immunoprecipitation

For immunoblots of immunoprecipitated (IP) lysate the following method was used. ID3 protein was pulled down using magnetic beads (Dynabeads protein G). In Brief, the lysate were incubated with 10 µg of antibody and 50 µl (1.5 mg) of Dynabeads (Invitrogen) in micro-centrifuge tubes with rotation for 10 minutes at room temperature. Samples were then placed on a magnetic particle concentrator (DynaMag™-2, Invitrogen) and supernatant was discarded. Dynabeads–Ab–Ag complex was washed 3 times with washing buffer and tubes were placed on magnetic particle concentrator to remove the supernatant. The magnetic Dynabeads–Ab–Ag complex was suspended with 20 µl of Elution Buffer and boiled in SDS sample buffer for 5 min. Samples were placed on the magnetic particle concentrator and supernatant containing protein were separated by SDS-PAGE and immunoblotted as described above.

### Phospho-peptide identification by (MALDI-TOF/TOF) mass spectrometry

The services of Applied Biomics (Hayward, CA, USA) were used for the identification of phosphorylation sites by MALDI-TOF/TOF following a standard protocol. In brief, after the PCB153 treatment total cell lysates were immunoprecipitated with ID3 and separated on a 1D gel. The gel bands corresponding to ID3 protein were reduced, alkylated, and subjected to trypsin digestion. Supel-Tips (Sigma-Aldrich) were used for phosphopeptide enrichment. Tryptic peptides were desalted by Zip-tip C18 (Millipore), and peptides were eluted from the Zip-tip with 0.5 µl of Matrix solution (Agilent Technologies) and spotted on the MALDI plate. MALDI-TOF MS (matrix-assisted laser desorption/ionization–time-of-flight MS) was performed on an AB Sciex Proteomics Analyzer (AB Sciex, Foster City, CA, USA). MS spectra were acquired in positive ion mode and ∼4000 laser shots/per spectrum. A virtual digest was done by submitting protein sequences of interest to University of California–San Francisco Protein Prospector (http://prospector.ucsf.edu/prospector/mshome.htm). The MS precursors matching the virtual digest were submitted for MS fragmentation. The resulting peptide masses were submitted to MASCOT search engine (Matrix Science) to search the database of Swiss-Prot. Candidates with either protein score C.I.% or ion C.I.% >95 were considered significant. The spectra of all peptides containing potential phosphorylation sites were manually evaluated for the loss of phosphate.

### In vitro kinase assay

FLAG-ID3 in vitro kinase assay: Active recombinant human Pyk2 kinase (0.1 µg/µl, Signal Chem) was added with ATP (10 mM, Signal Chem), ID3 protein (Recombinant protein with GST–Myc-DDK/FLAG, TP300583, OriGene), and incubated 37°C overnight. Samples were kept in 4°C for 10 min and boiled in SDS sample buffer for 5 min. Samples were seperated by 12% SDS-PAGE and immunoblotted as described previously. GST-ID3 in vitro kinase assay: Active recombinant human Pyk2 kinase (0.1 µg/µl, SignalChem) was added with ATP (10 mM, Signal Chem), with ID3 protein (Recombinant protein with GST–Tag, AAH03107, Abnova), and processed as described previously.

### ID3 overexpression

The hCMEC/D3 cells were stably transfected with with either Precision LentiORF for ID3 (Thermo Scientific Open Biosystems) or empty vector lentiviral pLEX-JRED/TurboGFP by the trans-lentiviral packaging kit with Express-in transfection reagent according to the manufacturer's instructions. We used the MOI (multiplicity of infection) of 25 and selected cells that overexpressed ID3 with blasticidin S (5 µg/ml) as per manufacturer's instructions. Cells expressing TurboGFP were identified by fluorescence microscopy.

### FITC Annexin V Apoptosis detection

Cell apoptosis was assessed by using FITC Annexin V Apoptosis Detection Kit I (BD Pharmingen) according to the manufacturer's protocol. Briefly, cells were washed with cold PBS and then resuspended in 1X binding buffer, and 100 µl of solution containing cells were stained with 5 µl Annexin V-FITC and 5 µl propidium iodide (PI). After incubation in the dark for 15 min, 100 µl of 1X binding buffer was added and cells were analyzed by Guava easyCyte using the CytoSoft software program according to the manufacturer's instructions.

## Results

### PCB-induced ROS and ID3 mediate neovascularization

**Figure 1:**
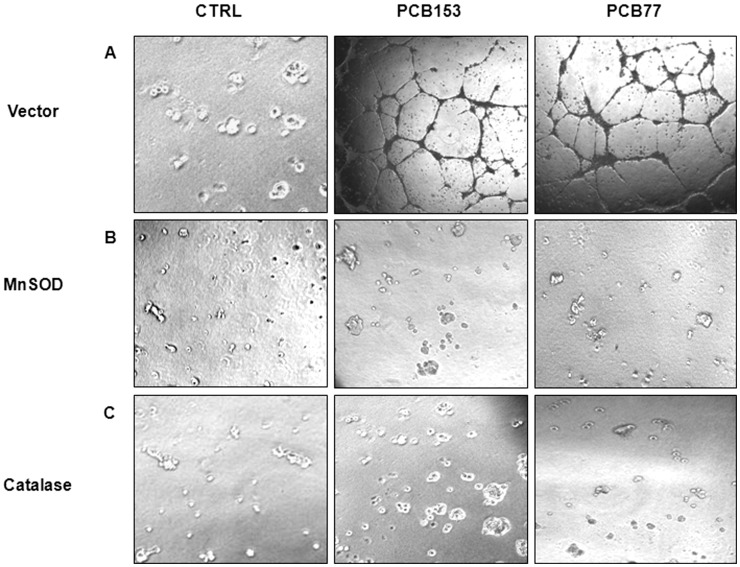
PCB-induced neovascularization phenotype depends on ROS. Representative micrographs showing inhibition of endothelial tube formation in HUVECs cultured in a matrix gel and exposed for 5(*Panel* ***A***). Co-treatments with antioxidant enzymes MnSOD (*Panel* ***B***) and catalase (*Panel* ***C***) were used to determine the contribution of specific ROS. HUVECs were treated with vehicle control (0.1% DMSO) or 1 ng/ml of PCB153 and PCB77 for 5 h. For antioxidant enzyme overexpression, prior to treatment HUVECs were incubated with replication deficient adenoviral constructs containing human MnSOD, catalase, or empty vector (ViraQuest Inc.) at 50 multiplicities of infection (MOI) for 24 h. HUVECs were treated with 1 ng/ml of PCB153 (2,2′,4,4′,5,5′-hexachlorobiphenyl) or PCB77 (3,3′,4,4′-tetrachlorobiphenyl).

ECs acquire a proliferative and invasive phenotype in the neovascular process; and there is some evidence that disorders such as microvascular lesion formation exploit this phenotype. Development of microvascular lesions has been described mechanistically as a neovascular process gone awry [4], [20]. The concept of oxidative stress or the excess of ROS that triggers the development of vascular disease has been implicated as a mechanism by which environmental pollutants may exert their adverse effects. Several studies have demonstrated that PCBs can induce oxidative stress in human vascular ECs [21]–[25]. Thus, it is biologically plausible that ECs that line the lumen of blood vessels are under increased oxidative stress from exposure to PCBs. Various PCB congeners have been shown to exert neovascular actions in cells. Therefore, we used the endothelial tube assay to determine whether PCB-induced neovascularization depended on ROS. Human umbilical vein endothelial cells (HUVECs) were cultured on top of Cultrex Reduced Growth Factor Matrix. In Panel A of Figure 1, a 5 h exposure to both PCB congeners 153 and 77 [1 ng/ml] resulted in a well developed tube network compared to vehicle control (0.1% DMSO). Next, we ascertained whether the individual ROS relevant to mediating this PCB-induced phenotype was either superoxide radical (O_2_ **•^––^**) or hydrogen peroxide (H_2_O_2_) by overexpressing ROS scavenging enzymes. NADPH oxidase has been identified as a source of O_2_ **•^––^** production in PCB exposed ECs [26]. To determine whether PCB-induced tube formation depended on O_2_ **•^–^**, HUVECs were transduced with an adenoviral vector expressing the enzyme MnSOD (*Ad-MnSOD*) which is capable of dismutating O_2_ **•^––^**. As shown in Panel B of Figure 1, overexpression of MnSOD inhibited tube formation and resulted in rounded EC colonies with few branch points in PCB treated cells compared to the vector control. Studies have shown that selectively overproducing or removing H_2_O_2_ significantly altered vascular lesion formation in animal models [27]. Thus, we determined whether H_2_O_2_ contributed to PCB-induced tube formation by overexpressing the H_2_O_2_ scavenging enzyme catalase. HUVECs were transduced with *Ad-CAT*. As shown in Panel C of Figure 1, overexpression of catalase similarly inhibited the PCB-induced endothelial tube network.

**Figure 2:**
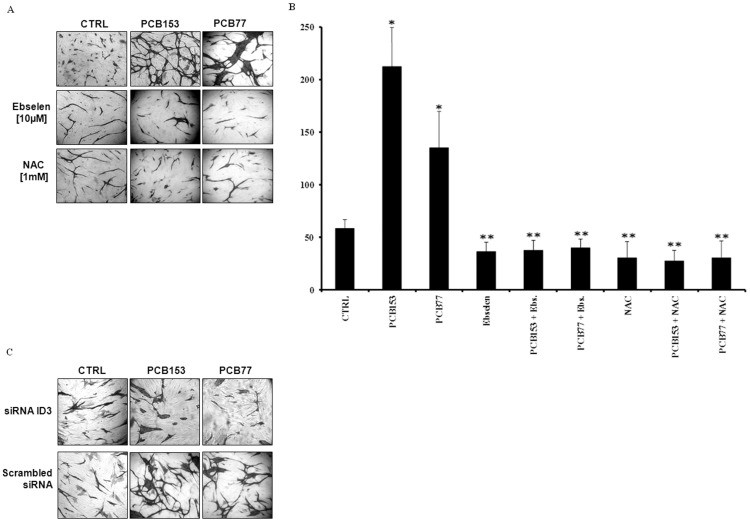
ROS scavengers and siRNA ID3 inhibit the PCB-induced neovascular phenotype. PCB-induced vessel formation was further characterized in a co-culture model. HUVECs were seeded on top of a confluent layer of fibroblasts (Fb). (**A**) Representative micrographs showing inhibition of endothelial tube formation. Cells were pretreated with ROS scavengers: ebselen (Ebs.) or N-acetylcysteine (NAC) for 2 h prior to treatment with 1 ng/ml of PCB153 and PCB77. Immunostaining for the endothelial marker CD31 at day 3 showed significant tube formation in the PCB treatment groups compared to vehicle control (0.1% DMSO) that was suppressed by ebselen and NAC treatment. (**B**) The effect of ROS scavengers on PCB-induced endothelial tube formation was quantified by counting the number of branch points. Co-cultures were established in triplicate and data are expressed as mean ± S.D. between different wells. Measurements were performed from a representative experiment repeated three times in triplicate. (*) PCB treatment significantly different from vehicle control. (**) ROS scavenger treatment significantly different from PCB (*P*<0.05). (**C**) ID3 siRNA treatments inhibit PCB—induced tube formation in HUVECs co-cultured with fibroblasts at 3 days. Immunostaining for the endothelial cell marker CD31 was used to visualize vessel formation. HUVECs were transfected with ID3 siRNA [50 nM] for 48 h prior to co-culture experiments. Scrambled siRNA (Silencer negative control siRNA, Ambion) was used in all RNAi experiments. The data shown are representative images from an experiment repeated three separate times.

Although endothelial tube assays performed on matrix gel are commonly used to determine EC vessel formation *in vitro*, Donovan et al. [28] have demonstrated that tube formation on a matrix gel is not specific for ECs. Moreover, they reported that endothelial vessel morphology from co-cultured cells better represents EC sprouting that occurs *in vivo*. Hence, we further verified the contribution of ROS in PCB-induced neovascular formation with the co-culture assay. HUVECs were seeded on top of a confluent layer of fibroblasts and treated with PCB153, PCB77, ROS scavengers, or the vehicle control (DMSO) for 72 h. Cell cultures were immunostained for endothelial marker CD31 and showed significant tube networks in both PCB153 and PCB77 treated cells compared to control (Fig. 2A). Pretreatment with the ROS scavengers, ebselen and N-acetylcysteine (NAC) resulted in EC morphology of short tubes with significantly fewer branches than with the PCB treatments alone. Inhibition of PCB-induced neovascular phenotype by ROS modifiers was quantified by counting the mean number of branch points. Results are shown in Fig. 2B where the quantification of branch points was evaluated in micrographs from four fields of each well. Our PCB-induced endothelial phenotype changes shown in both the matrix gel and co-culture EC tube assays confirmed that ROS support the development of a significant neovascular network in HUVECs exposed to both the non-dioxin-like PCB153 and dioxin-like PCB77. Although a previous study reported significant ROS production from exposure to PCB77, but not with PCB153 [25]; the conditions used in our experiments were not similar. Our experimental conditions differed in that we used a human EC model and a far lower dose of PCB153. These major differences in conditions can account for the observed ROS dependent tube formation seen in the PCB153 treatment. In further support of our data, a more recent study reported that human brain ECs exposed to 5 µM of PCB153 significantly produced ROS [26].

ID3 has been implicated in vascular lesion formation in response to injury and in native atherosclerosis [29]. PCB exposure is known to induce ROS in ECs and since ID3 is regulated by the redox status of the cell, we determined whether ID3 was critical for the observed PCB-induced neovascular phenotype. To our knowledge, the effects of PCBs on Id proteins in ECs have not been previously studied. Therefore, cellular levels of ID3 were knocked down by siRNA and phenotype changes were determined with the co-culture tube assay as described previously. HUVECs were transfected with ID3 siRNA [50 nM] or Silencer negative control siRNA (Ambion) for 48 h prior to co-culture experiments. Cells were treated with PCB153 and PCB77 as described previously for 72 h. Immunostaining for the endothelial marker CD31 was used to visualize vessel formation. In Fig. 2C, ID3 siRNA treatment showed a significant reduction in PCB-induced vessel formation by both PCB congeners 153 and 77 compared to the negative control. The inhibition of neovascularization by ROS scavengers and siRNA knockdown of ID3, suggest that PCBs exert these changes in EC phenotype by ROS-signaling pathways.

### PCB153-induced endothelial spheroid formation

Recent data from our laboratory showed that ID3 induced a molecular stem cell-like signature—CD133^+^ VEGFR3^+^ CD34^+^ in microvascular ECs (*in press*). Even though blood vessels are lined by a single layer of ECs, cells in vascular lesions do not follow the “law of the monolayer.” Rather than growing in a flat monolayer, the ECs grow in three dimensions similar to tumors. Spheroid formation is known to correlate with cell survival or increased resistance to apoptosis. Since hyper-proliferative, apoptosis-resistant, and monoclonal ECs have been characterized in microvascular lesions [30]; we used a stem cell sphere forming assay to determine the influence of PCB153 and ROS on the formation of endothelial spheroids.

**Figure 3:**
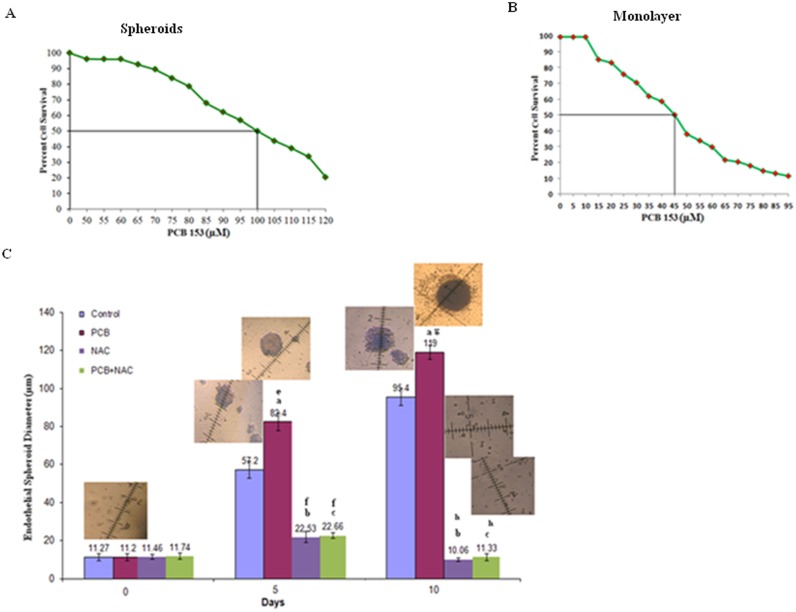
PCB153 treatment increased the survival and size of endothelial spheroids. (**A**) Graph of cell survival from exposure to PCB153 in B27 media determined by MTT assay of endothelial spheroids. Cell survival was determined 24 h after exposure. LC_50_∼100 µM. Results shown are averages of triplicates ± SD (<10%). (**B**) Graph of cell survival from exposure to PCB153 in monolayer culture in routine culture media determined by MTT assay. Cell survival was determined 24 h after exposure to PCB153. LC_50_∼45 µM. Results shown are averages of triplicates ± SD (<10%). (**C**) PCB153 (60 µM) treatment increased the diameter of endothelial spheroids measured at both days 5 and 10. The hCMEC/D3 cell line was cultured with serum free B27 medium in ultra-low attachment 96 well plates. Spheroid diameter was measured at days 5 and 10. The measurement scale was 100 µm at a magnification of 200×. Error bars represent the mean diameter of 15 spheroids ± SD. Data were analyzed by ANOVA; Tukey HSD test for multiple comparisons. Endothelial spheroid size is the mean diameter of 15 spheroids per treatment group ± SD. ^a^p<0.01; ^b^p<0.01; ^c^p<0.01 vs control within 5 and 10 days as well as ^e^p<0.01; ^f^p<0.05 vs control between 0 and 5 days and ^g^p<0.01; ^h^p<0.01 vs control between 0 and 10 days. Representative microphotographs of treatment groups are shown inset.

The non-dioxin-like PCB congener 153 accounts for 15–30% of total PCB content in most human samples and highly ortho-cholorine-substituted PCBs like PCB153 have been reported to accumulate in the brain [31]. We continued our experiments using the immortalized human cerebral microvascular endothelial cell line hCMEC/D3. PCB153 treated hCMEC/D3 have already been shown to produce ROS [26], hence we further determined whether PCB153 or ROS could support the growth of EC spheroids. The effect of a single dose of PCB153 on the survival of hCMEC/D3 cultured in non-adherent round bottom plates and B27 supplemented DMEM/F12 media was assessed for 24 h. Under these conditions stem-like cells were subjected to the MTT assay and cell viability was determined. PCB153 inhibited hCMEC/D3 cells with 50% survival (LC_50_) at 100 µM (Fig. 3A). Therefore, we treated hCMEC/D3 cells to a single dose of 60 µM of PCB153 for 10 days. It is important to note that toxicity of PCB153 exposed hCMEC/D3 differed greatly when cells were exposed in routine monolayer culture conditions. In monolayer culture, the LC_50_ was 45 µM (Fig. 3B) which is to be expected because cells in monolayer are composed of a heterogeneous population of quiescent, progenitor, senescent, and dead cells that may be less tolerant to PCB exposure when compared to more resilient stem-like cells that are selected for by the sphere assay. Using the stem cell sphere assay described previously, ECs were treated with PCB153 to determine whether this exposure selected for the formation of endothelial spheroids. Measurements of the diameters of individual endothelial spheres were taken at days 0, 5, and 10. As shown in Fig. 3C, PCB153 treatment resulted in significantly larger endothelial spheroids at both days 5 and 10 compared to vehicle control (0.1% DMSO). In vehicle control, the stem cell sphere assay selected for the survival and growth of stem-like endothelial colonies that was enhanced by PCB153 treatment. The majority of research on PCB-induced ROS has focused on their cellular toxicities. Based on our previous data showing ROS scavengers inhibited PCB-induced neovascularization, hCMEC/D3 cells were pre-treated for 1 h with 3 mM of NAC prior to PCB153 treatment on the day of cell seeding. Our data showed that NAC significantly inhibited the size of PCB153-induced endothelial spheroids in both the vehicle control and PCB153 groups. The significant reduction in the size of endothelial spheroids by NAC treatment alone when compared to control showed that endogenous levels of ROS contributed to sphere formation and cell survival. This importance of endogenous ROS levels was not surprising because clonal stem cell sphere formation and self-renewal have been reported to depend on high endogenous ROS levels [32]. Based on our data, the individual contributions of ROS from cellular metabolism or PCB exposure to spheroid formation cannot be distinguished. However, the findings that showed PCB153 increased the size of endothelial spheroids and survival is novel. Even though the sphere forming assay is widely used to select for stem-like cells under serum-free culture conditions, this is the first report to our knowledge that included exposure to environmental PCBs.

### ID3 expression correlated with benign and malignant cardiovascular tissues

**Figure 4:**
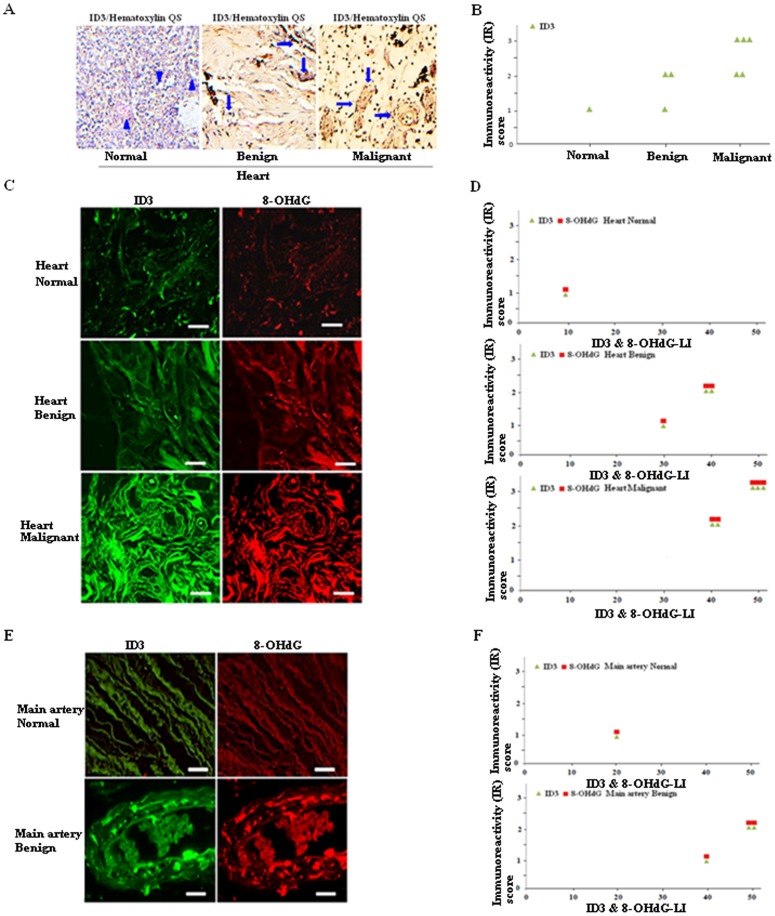
ID3 protein and oxidative damage is highly expressed in benign and malignant cardiovascular tissues. (**A**) Representative immunohistochemical analysis of heart tissue using ID3 rabbit monoclonal antibody. ECs from normal tissue did not exhibit detectable amounts of ID3 protein (arrowhead) while (arrows) indicated brown color as highest expression level for ECs with ID3 in vessels from benign and malignant heart tissue. Counter-stained with Hematoxylin QS. Magnification 200×. (**B**) Scatter plot of immunoreactivity (IR) score vs. ID3 in normal, benign, malignant heart. Spearman's rank correlation test showed a significant correlation between ID3 immunostaining and degree of malignancy with the ID3 IR scores significantly higher in benign compared to normal (p = 0.04) and in malignant compared to normal (p = 0.02). (**C**) Immunofluorescence staining of ID3 (green) and 8-OHdG (red) in heart tissue. Scale = 20 µm. (**D**) Scatter plot of immunofluorescence (IF) score vs ID3 & 8-OHdG labeling index (LI) in normal, benign, and malignant heart. IF score represents total score taken as the product of fluorescence intensity score and the cell score. Labeling index (LI) represents the percentage of the immunostained cells per total number of cells. Corresponding graphs to each row of images indicate a higher ID3 & 8-OHdG LI in both malignant and benign tissues compared to normal tissue (p<0.05, Mann-Whitney U-test). (E), Immunofluorescence staining of Id3 (green) and 8-OHdG (red) in main artery tissue. Scale = 20 µm. (F) Scatter plot of IF score vs ID3 & 8-OHdG LI in benign and normal artery tissue. IF score represents total score taken as the product of fluorescence intensity score and the cell score.

Id proteins are positive regulators of cell growth and play a major role in neovascularization. High levels of ID3 mRNA expression have been reported in the rat carotid injury model [14]. It is not clear whether ID3 mRNA levels are a reliable marker of phenotyping microvascular lesion formation. Based on our findings that showed siRNA ID3 suppressed PCB-induced neovascularization, we first performed immunohistochemical microscopy of ID3 protein expression in a tissue microarray of malignant, benign, and normal human vascular tissue specimens. We used a cardiovascular disease tissue microarray (US Biomax) that was designed for blood vessel or EC related studies. Immunostaining of vessels with ID3 was semi-quantitatively evaluated by assigning a score for the intensity of the immunohistochemical reaction and for the proportion of the ID3 cells stained. The product of these two values was taken to give the overall immunoreactivity (IR) score. The intensity of the immunohistochemical reaction (intensity score) was stratified into four categories: 0, no IR; 1, weak IR; 2, moderate IR; and 3, strong IR. In adult normal heart, ECs did not exhibit detectable amounts of ID3 protein (arrowhead) Fig. 4A. In contrast, strong immunostaining was detected for ID3 in vessels from benign and malignant heart specimens (arrows). Slides were counter-stained with hematoxylin QS. In Fig. 4A, blood vessels (arrows) in malignant heart tissue showed thickened walls with partially occluded lumens and robust ID3 staining of ECs that lined the lumen compared to the lumens found in the benign heart vessels (arrows). Fig. 4B showed the ID3 IR score distribution in heart specimens with different degrees of histological malignancy. Spearman's rank correlation test showed a significant correlation between ID3 immunostaining and degree of malignancy with the ID3 IR scores significantly higher in benign compared to normal (p = 0.04) and in malignant compared to normal (p = 0.02). Since the regulation of ID3 expression is known to depend on cellular redox status, we further determined whether the protein levels of ID3 and a marker of oxidative DNA damage, 8-hydroxydeoxyguanosine (8-OHdG), correlated with the degree of malignancy. In Fig. 4C, immunofluorescence staining of ID3 (green) and 8-OHdG (red) in the malignant, benign, and normal human heart vascular tissue specimens are shown. As described previously, immunoreactivity scores for ID3 and 8-OHdG were semi-quantitatively determined and reported in scatter plots of IR scores vs. ID3 & 8-OHdG labeling index for each degree of histological malignancy (Fig. 4D). Labeling index (LI) represents the percentage of the immunostained cells per total number of cells. We observed a higher ID3 & 8-OHdG LI in both malignant and benign tissues compared to normal tissue (p<0.05, Mann-Whitney U-test). In Fig. 4E, immunofluorescence staining of Id3 (green) and 8-OHdG (red) are shown in main artery tissue. ID3 & 8-OHdG LI for benign are greater than normal artery tissue (Fig. 4F; p<0.05, Mann-Whitney U-test). In summary, the findings from the tissue microarray showed that a higher expression of ID3 protein correlated with a greater degree of malignancy in blood vessels of heart tissue. The mechanism accounting for this perturbation of ID3 expression under different degrees of histological malignancy is not known, however, our immunofluorescence data showed a correlation between oxidative DNA damage marker 8-OHdG and ID3.

### PCB153-induced ID3 expression depends on ROS but not on AhR signaling

**Figure 5:**
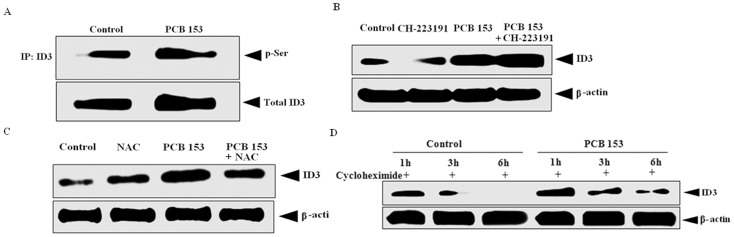
PCB-induced ID3 expression is independent of AhR and ID3 protein is stablized. (**A**) PCB153 [5 µM] treatment increased serine phosphorylation of ID3 protein at 3 h. ID3 protein was isolated by immunoprecipitation (IP) technique using magnetic beads and detected by immunoblot. Serine phosphorylation of immuoprecipitated ID3 protein was determined with anti-p-Ser antibody. (**B**) PCB153 induced ID3 protein was not modulated by aryl hydrocarbon receptor (AhR) at 3 h in hCMEC/D3 cell line. Western blot analysis showed that the AhR ligand-selective antagonist, CH-223191 [10 nM], did not prevent PCB153 induced expression of ID3. (**C**) PCB153 induced expression of ID3 protein was inhibited by ROS scavenger NAC in hCMEC/D3. (**D**) PCB153-treated cells in the absence of protein synthesis showed that ID3 protein levels remained higher than control at 3 and 6 h time points. Cells were pretreated with the protein synthesis inhibitor cycloheximide [1 µg/ml] 2 h prior to treatment with PCB153.

Vascular cell growth has been shown to be regulated by serine phosphorylated ID3 [33]. We previously have shown that estradiol exposure to HUVECs increased the serine phosphorylation of ID3 via ROS. Although our earlier experiments show that ID3 siRNA inhibited PCB-induced neovascularization of HUVECs, the effect of PCB153 exposure has on ID3 in the microvascular cell line hCMEC/D3 was not known. Therefore, we determined whether hCMEC/D3 exposed to PCB153 showed increased ID3 serine phosphorylation (p-Ser) and expression. As shown in Fig. 5A, PCB153 exposure significantly increased the levels of p-Ser ID3 and total ID3 protein expression at 3 h. PCBs have been reported to exert their toxicity through binding to the aryl hydrocarbon (dioxin) receptor (AhR) which is a ligand-dependent transcription factor. Although the AhR binding affinity of non-dioxin-like PCB congener 153 is relatively weak compared to the AhR ligand TCDD, PCB153 has been reported to bind the AhR at high concentrations [34]. Therefore, we determined whether PCB153-induced ID3 expression in ECs depended on AhR signaling by treating with a known AhR ligand-selective antagonist, CH-223191 [35]. Co-treatment with the CH-223191 [10 nM] did not interfere with the PCB153-induced increase in ID3 protein (Fig. 5B). These findings demonstrate that PCB153-induced increase in p-Ser ID3 and total ID3 was not mediated via AhR signaling. We and others have reported that ID3 expression is ROS dependent in ECs. To investigate whether PCB153-induced ROS mediated ID3 expression in hCMEC/D3, we pre-treated ECs with the ROS scavenger N-acetylcysteine. As shown in Fig. 5C, the treatment with N-acetylcysteine inhibited the PCB153-induced increase of ID3 expression at 3 h in hCMEC/D3. Because the level of ID3 protein is determined by the rates of protein synthesis and protein degradation, we tested whether PCB153 treatment affected the protein synthesis of ID3. As shown in Fig. 5D, ID3 protein decreased upon treatment with the protein synthesis inhibitor cycloheximide in the control. PCB153-treated cells in the absence of protein synthesis showed that ID3 protein levels remained higher than control at 3 and 6 h time points. Thus, the observed increase in ID3 by PCB153 treatment does not depend on protein synthesis in hCMEC/D3, but rather PCB153 made ID3 more stable from protein degradation.

### Identification of phosphorylated amino acids in microvascular endothelial ID3

**Figure 6:**
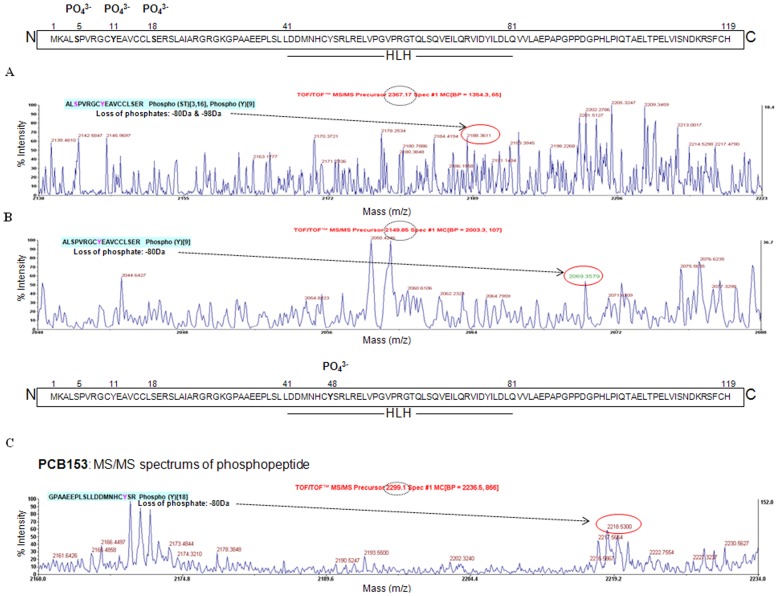
Identification of phosphorylated amino acids in ID3 from microvascular ECs. MALDI-TOF/TOF spectra for peptides extracted from hCMEC/D3 cells. Vehicle control showed that Ser-5 or Ser-18 (*Panel* ***A***) and Tyr-11 (*Panel* ***B***) are phosphorylated. Identification of phosphopeptides from PCB153 treated cells indicated that along with Ser-5 that was detected at the level of MS, Tyr-48 was also phosphorylated (*Panel* ***C***). Methods: Cell lysate was immunoprecipitated with ID3 antibody from vehicle control and PCB153 treated hCMEC/D3 cells. The immunoprecipitated lysate was separated by 1D-SDS-PAGE and the protein band corresponding to the MW of ID3 was excised from the gel, subjected to tryptic digestion and phosphopeptide enrichment. The phosphopeptides were spotted on a MALDI plate followed by MS and database search. The spectra of all peptides were manually evaluated for the loss of phosphate which is shown in red circles and the observed mass (dashed circle) and sequence of the peptides are shown. *X-axis* represents mass and *Y-axis* represents intensity.

Serine-5 phosphorylated ID3 has been shown to be important for cell growth [33], although it is not known whether this is also the case for microvascular ECs. Given that there is no commercially available antibody specific for Ser5-phosphorylated ID3, we performed a search for the presence of any literature-derived kinase/phosphatase motifs in the human ID3 sequence using the PhosphoMotif Finder program. This software program contains experimentally characterized phosphorylation-based substrate and binding motifs derived from the literature and has been integrated with Human Protein Reference Database (HPRD—http://www.hprd.org/ [36]. A search with the PhosphoMotif Finder program revealed that ID3 had 38 serine kinase/phosphatase motifs and 8 tyrosine kinase/phosphatase motifs (Table S1 and S2 in File S1) described in the literature. Based on this information, PCB153 treatment has the potential to induce several serine and/or tyrosine phosphorylation sites. To determine phospho-ID3 sites induced by PCB153, cell lysates from treated hCMEC/D3 were immunoprecipitated with ID3 and prepared for MALDI-TOF analysis as described in Materials & Methods section. MS spectra analysis identified with high confidence the human ID3 sequence with the protein score of 99% from the control sample and 94% from the PCB153 sample. Presence of phosphorylated residues was identified by the MS/MS peak showing neutral loss of phosphates. In control, we observed a peptide AL**S** ^5^ PVRGC **Y** ^11^ EAVCCL **S** ^18^ER, m/z 2367.1738, which corresponded to the calculated mass of the loss of phosphate (−98 Da for serine or theronine phosphorylated peptides) plus loss of phosphate (−80 Da for tyrosine phosphorylated peptide) (Fig. 6A). We also observed a peptide ALSPVRGC **Y** ^11^ EAVCCLSER, m/z 2149.8459, which corresponded to the calculated loss of phosphate (−80 Da) (Fig. 6B). In the PCB153 treatment group, along with peptides where Ser-5 was detected at the level of MS, we observed a peptide GPAAEEPLSLLDDMNHC **Y** ^48^SR, m/z 2298.0977, which corresponded to the calculated loss of phosphate (−80 Da) for a tyrosine phosphorylated peptide (Fig. 6C). Thus, the peptide spectra data demonstrated that multiple amino acids were phosphorylated on ID3. These phosphorylated amino acids included either Ser-5 or Ser-18 in combination with Tyr-11 as observed in the control while an additional Tyr-48 residue was identified in the PCB153 treatment group.

### PCB153 increased tyrosine phosphorylation of ID3

**Figure 7:**
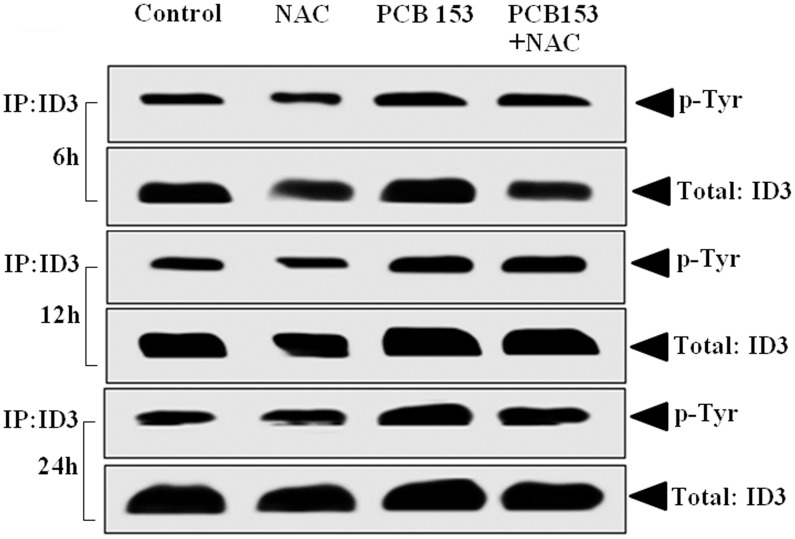
PCB153 increased tyrosine phosphorylation of ID3. hCMEC/D3 cells were treated with PCB153 to determine its effect on the tyrosine phosphorylation state of ID3 at 6, 12, and 24 h. After 24 h of PCB153 [10 µM] treatment, we observed a significantly higher level of tyrosine phosphorylation than vehicle control. The phospho-Tyr bands detected from immunoprecipitated (IP) cell lysate corresponded with the molecular weight (MW) of 17 kDa for ID3. IP was performed to isolate ID3 from cell lysate using a monoclonal anti-ID3 antibody. Magnetic bead-based separation was then used to extract the bound ID3 protein via Protein G coupled to superparamagnetic Dynabeads. P-Tyr was detected by immunoblotting using the anti-phospho-Tyr antibody. Electrochemiluminescence (ECL) band intensity for phospho-Tyr was imaged with a Bio-Rad Versa Doc instrument.

Although phosphorylated Ser-5 of ID3 has been reported, we have not found any evidence from the literature of tyrosine phosphorylated ID3. Based on our search results from PhosphoMotif Finder, it revealed that the ID3 protein sequences at Tyr-11 and Tyr-48 corresponded to Src, JAK2, and ALK kinase substrate motifs. We further confirmed whether PCB153-induced tyrosine phosphorylation of ID3 protein in the hCMEC/D3 cell line. Immunoprecipitation (IP) was performed to isolate ID3 from cell lysate using a monoclonal anti-ID3 antibody (Cal-Bioreagent). Magnetic bead-based separation was then used to extract the bound ID3 protein via Protein G coupled to superparamagnetic Dynabeads according to the manufacturer's instructions. Proteins from immunolysates were separated by SDS-PAGE, transferred to PVDF membrane, and processed as described previously. Western Blots with phosphorylated ID3 were detected by electrochemiluminescence (ECL). The ECL band intensity for phospho-Tyr was imaged with a Bio-Rad Versa Doc instrument. ECs were treated with PCB153 to determine its effect on the tyrosine phosphorylation state of ID3 at 6, 12, and 24 h. After 24 h of PCB153 [10 µM] treatment, we observed a significantly higher level of tyrosine phosphorylation than vehicle control (Fig. 7). The phospho-Tyr bands corresponded with the molecular weight (MW) of 17 kDa for ID3. Co-treatment with a ROS scavenger was used to determine whether it would change the level of PCB-induced tyrosine phosphorylation, however, we did not observe a significant effect by N-acetylcysteine (2 mM). These findings showed that PCB153 treatment increased tyrosine phosphorylation of immunoprecipitated ID3 lysate and confirmed the MS/MS spectra data that showed phosphorylated amino acids Tyr-11 and Tyr-48 in peptides from ID3.

### Pyk2 mediated PCB-induced neovascularization and ID3 protein levels

**Figure 8:**
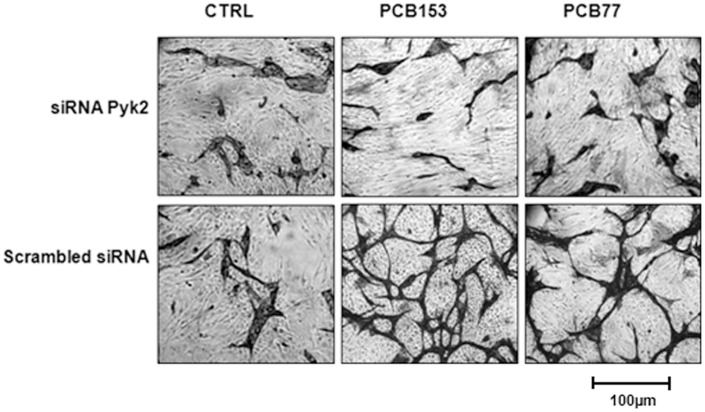
siRNA Pyk2 inhibited the PCB-induced neovascular phenotype. PCB-induced vessel formation was characterized in a co-culture model in which HUVECs were seeded on top of a confluent layer of fibroblasts (Fb). HUVECs were transiently transfected with either Pyk2 siRNA [50 nM] or Silencer negative control siRNA for 48 h prior to experiments. Co-cultures immunostained for endothelial marker CD31 showed a robust inhibition of PCB-induced neovascular network by Pyk2 siRNA when compared to cells transfected with negative control siRNA. Scrambled siRNA (Silencer negative control siRNA, Ambion) was used in all RNAi experiments. The data shown are representative images from an experiment repeated three separate times. HUVECs were treated with 1 ng/ml of PCB153 (2,2′,4,4′,5,5′-hexachlorobiphenyl) or PCB77 (3,3′,4,4′-tetrachlorobiphenyl).

Many environmental toxicants exhibit their adverse effects via ROS signaling. Proline-rich tyrosine kinase 2 (Pyk2) represents a novel member of the focal adhesion kinase family that is activated by ROS in vascular cells [37]. Since the tyrosine kinase Src was reported to regulate both Id transcript and protein levels [38], we postulated that ID3 may be a novel target of Pyk2 signaling. We first determined whether Pyk2 was a positive regulator of the PCB-induced vascular phenotype by siRNA experiments as described previously. Cells were transfected with either Pyk2 siRNA or Silencer negative control prior to co-culture experiments. As shown in Fig. 8, ECs immunostained with CD31 showed a dramatic inhibition of both PCB153— and PCB77— induced tube formation by Pyk2 siRNA. These findings demonstrated that PCB-induced neovascular phenotype depends on Pyk2.

**Figure 9:**
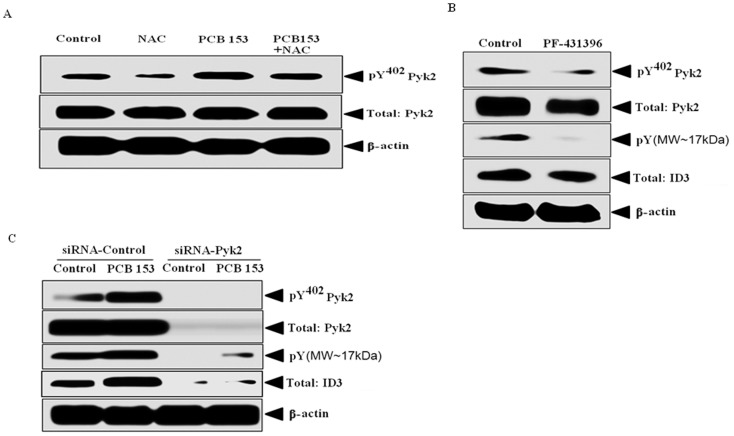
PCB153 increased Pyk2 phosphorylation & Pyk2 regulated ID3 protein. (**A**) hCMEC/D3 cells were treated with PCB153 to determine its effect on Pyk2 phosphorylation and whether the effect depended on ROS. PCB153-induced ROS increased the level of pY^402^ Pyk2 which was inhibited by the ROS scavenger N-acetylcysteine. (**B**) To determine whether ID3 was a downstream target of functional Pyk2 kinase, ECs were treated with the chemical Pyk2 kinase inhibitor PF-431396 [1 µM]. Pyk2 inhibitor inhibited the level of pY^402^ Pyk2, but did not change ID3 expression compared to vehicle control. (**C**) Using a loss-of-function approach with siRNA Pyk2, we compared the effects of inhibiting Pyk2 on the total level of ID3 protein. Using Lipofectamine RNAiMAX reagent (Invitrogen) ECs were transfected with either Pyk2 siRNA or Silencer negative control siRNA for 48 h prior to PCB153 treatments. Pyk2 siRNA inhibited total ID3 protein levels in both the PCB153 and vehicle control treated hCMEC/D3 cells compared to the negative control. Pyk2 siRNA treatment also inhibited the level of phosphotyrosine that corresponded to MW∼17 kDa of ID3.

The activation of Pyk2 results from its autophosphorylation on Tyr-402 (pY^402^), which creates a Src homology 2-binding site (SH2). Next, we determined whether PCB153 modulated Pyk2 phosphorylation in hCMEC/D3 cells. The aim of this experiment was to determine whether PCB153-induced ROS increased the level of pY^402^ Pyk2. Cells pretreated with the ROS scavenger N-acetylcysteine (Fig. 9A), showed significantly less PCB153-induced Pyk2 phosphorylation then in the control panel. There have been no reports to our knowledge of Pyk2 mediated ID3 therefore we determined whether ID3 was a downstream effector of Pyk2 kinase. Using a loss-of-function approach with both Pyk2 kinase inhibitor (PF431396) and siRNA, we compared the effects of inhibiting Pyk2 on the total level of ID3 protein. To determine whether ID3 was a downstream target of functional Pyk2 kinase, ECs were treated with the chemical Pyk2 kinase inhibitor PF-431396. As shown in Fig. 9B, exposure to the Pyk2 inhibitor attenuated the level of pY^402^ Pyk2, however, we did not observe any significant change in the expression of ID3 compared to vehicle control. Pyk2 is known to phosphorylate tyrosine residues, but because there are no commercially available anti-phosphotyrosine-ID3 antibodies, we determined the effect of PF-431396 treatment on the level of phospho-tyrosine at a MW of approximately 17 kDa by stripping and re-probing ID3 immunoblots with anti-phosphotyrosine antibody. As expected, we observed a significant decrease in the intensity of the phospho-tyrosine band in cells treated with PF-431396 (Fig. 9B). Since chemical inhibitors may exert non-specific effects, we compared the observations from the PF-431396 treatment to siRNA experiments. ECs were transfected with either Pyk2 siRNA or Silencer negative control siRNA for 48 h prior to PCB153 treatments. As shown in Fig. 9C, Pyk2 siRNA significantly decreased the total ID3 protein levels in the PCB153 treated ECs when compared to cells transfected with the scrambled siRNA control. Pyk2 siRNA treatment also inhibited the basal expression of ID3 in the vehicle control and the level of phosphotyrosine that corresponded to MW∼17 kDa of ID3. Taken together these findings demonstrated that Pyk2 mediates the expression of ID3 protein. In addition, the observed PCB153-induced increase in the level of tyrosine phosphorylation for proteins of MW∼17 kDa implied that ID3 may be a target of Pyk2 kinase.

### Pyk2 phosphorylated ID3 in vitro

**Figure 10:**
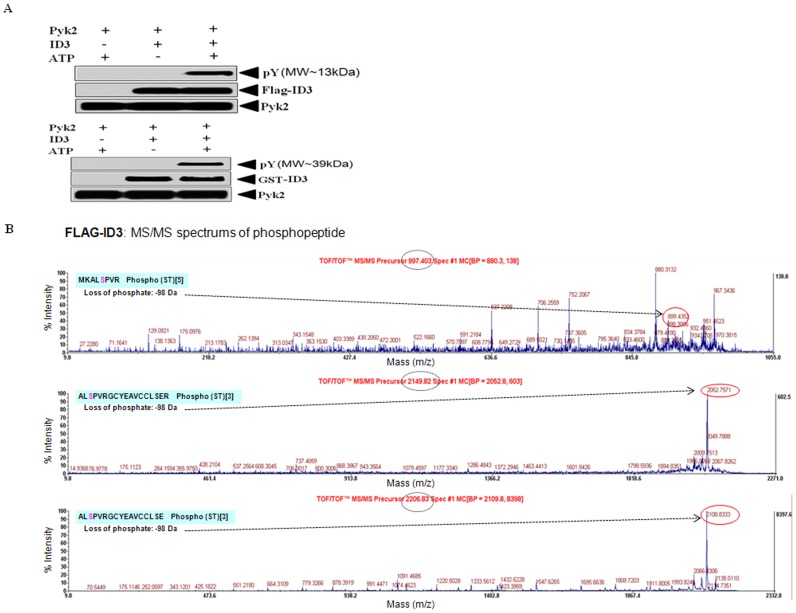
Recombinant active Pyk2 kinase phosphorylated ID3. Pyk2 siRNA significantly inhibited tyrosine phosphorylation for proteins of MW∼17 kDa corresponding to ID3 therefore we determined whether ID3 was phosphorylated by recombinant active Pyk2 in an *in vitro* kinase assay. Pyk2 kinase was incubated with either Flag-tagged ID3 or GST-tagged ID3 in the presence or absence of ATP; and phosphorylation was detected by immunoblotting. (**A**) A phosphorylated tyrosine band was detected at the corresponding MW∼13 kDA for FLAG-ID3 and MW∼39 kDa for GST-ID3. (**B**) Phosphorylation of in vitro–phosphorylated FLAG-ID3 was confirmed by combined MS+MS/MS as described previously. MS spectra analysis identified the human ID3 sequence with the protein score of 100%. Presence of phosphorylated amino acids was identified by the MS/MS peak showing neutral loss of phosphates which is shown in red circles and the observed mass (dashed circle). The sequence of the peptides are shown. *X-axis* represents mass and *Y-axis* represents intensity.

Since our data from the previous experiments showed that Pyk2 siRNA significantly inhibited the levels of ID3 and tyrosine phosphorylation for proteins of MW∼17 kDa corresponding to ID3, we postulated that Pyk2 phosphorylated ID3. Recombinant activated Pyk2 kinase was incubated with either Flag-tagged ID3 or GST-tagged ID3 in the presence or absence of ATP; and phosphorylation was detected by immunoblotting as described in the Materials & Methods section. As shown in Fig. 10A, we detected a phosphorylated tyrosine band at the corresponding MW∼13 kDA for Flag-ID3 and MW∼39 kDa for GST-ID3. Since the GST-tag from the recombinant ID3 protein may lead to inaccurate conclusions because the tag itself has been reported to be phosphorylated by kinase [39], we further confirmed Flag-ID3 phosphorylation status by MALDI-TOF/TOF. To identify the ID3 amino acid residue (or residues) phosphorylated by Pyk2, we analyzed in vitro–phosphorylated ID3 by combined MS+MS/MS as described previously. MS spectra analysis identified with high confidence the human ID3 sequence with the protein score of 100%. Presence of phosphorylated amino acids was identified by the MS/MS peak showing neutral loss of phosphates. As shown in Fig. 10B, we observed three separate phosphopeptide fragments: MKAL**S^5^**PVR, m/z 997.403; AL**S** ^5^ PVRGCYEAVCCLSER, m/z 2149.8193; and AL**S** ^5^ PVRGCYEAVCCLSE, m/z 2206.8262 that corresponded to the calculated mass of the loss of phosphate (−98 Da for serine phosphorylated peptides). Although Pyk2 kinase has not been shown *in vivo* to phosphorylate serine residues, our data indicated Ser-5 of Flag-tagged ID3 was phosphorylated under the conditions of the kinase assay. However, at the level of MS peptide fragments phosphorylated at Tyr-11 and Tyr-48 residues corresponding to the calculated mass of phosphorylated fragments: GCY^11^ EAVCCLSER, m/z 1412.5156 and GPAAEEPLSLLDDMNHCY ^48^SR, m/z 2298.0593, respectively were observed. Together these findings from the combined MS+MS/MS data confirm that Flag-tagged ID3 is phosphorylated by active recombinant Pyk2 kinase; and support the phospho-Tyr band that was detected at the corresponding MW∼13 kDA for Flag-ID3 by immunoblot.

### Stable ID3 overexpression increased cell survival of microvascular ECs

**Figure 11:**
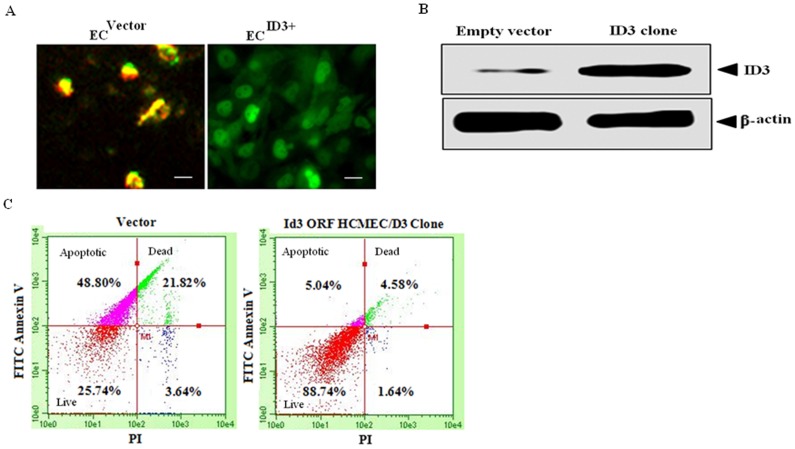
ID3 overexpression increased cell survival of ECs. The functional significance of ID3 on survival of hCMEC/D3 cells was determined by generating a stable ID3 overexpressing cell line. (**A**) and (**B**) Confirmation of ID3 overexpression in a stable clone of hCMEC/D3 cells was shown by both immunoflouorscence microscopy and immunoblot of ID3 levels. (**C**) Cells were subjected to FACS analysis after FITC Annexin V (*Y-axis*) and Propidium Iodide (PI: *X-axis*) staining. ID3 clone indicated a greater percentage of live cells stained negative for both FITC Annexin V and PI. ID3 clone showed 88.74% of cell population was not apoptotic compared to a far lower percentage of 25.74% live cells in the control.

Since our data indicated that PCB-induced neovascularization depended on ID3 and given the biological signficiance of ID3 as a positive regulator of cell growth, we asked whether stable overexpression of ID3 could increase cell survival of microvascular ECs. To address this question, adult human microvascular ECs were transduced using a MOI of 25 with either the Precision LentiORF for ID3 overexpression or control empty lentiviral vector pLEX-JRED/TurboGFP according to the manufacturer's instructions. Cells were then seeded at clonal density and selected with blasticidin S (5 µg/ml). Although other studies have reported ID3 overexpression in various cell lines, this is the first report of a stable ID3 clone in human cerebral microvascular endothelial cell line hCMEC/D3. As shown in Fig. 11A, fluorescent microscopy showed positive confirmation of TurboGFP expression for the stable ID3 clone while the control cells transduced with the empty vector express RFP and GFP. We next determined whether the protein expression level of ID3 in our stable clone was indeed greater than in the control. We detected a significantly higher level of ID3 protein in the stable ID3 clone compared to the empty vector control (Fig. 11B). Next we determined the functional consequence of ID3 overexpression on the percentage of cells within the hCMEC/D3 population that were actively undergoing apoptosis. Cells were subjected to FACS analysis after FITC Annexin V (*Y-axis*) and Propidium Iodide (PI: *X-axis*) staining (Fig. 11C). Live cells stained negative for both FITC Annexin V and PI and indicated that 88.74% of cell population from the ID3 clone were not undergoing measurable apoptosis compared to only 25.74% in control. Cells which stained positive for FITC Annexin V and negative for PI indicated that only 5.04% of the ID3 clone population was undergoing apoptosis compared to 48.8% in the control. Dead cells that stained positive for both FITC Annecin V and PI were 4.58% of ID3 clone population and 21.82% in the control. Thus, our data showed a significant increase in cell survival by ID3 overexpression in hCMEC/D3 cells.

## Discussion

In the present study, we determined whether PCB-induced ROS contributed to neovascularization with the aim of elucidating the role of environmental PCBs in endothelial dysfunction while focused on ID3. Our study investigated the effects of PCBs on endothelial cells because they are the cellular interface between environmental toxicants in the circulating blood and the vessel wall; and hence a prime target for the adverse effects of these environmental toxicants. Enhanced neovascularization has been implicated in either the development or progression of proliferative lesions in diabetic retinopathy, plexiform lesions in idiopathic pulmonary arterial hypertension, and atherosclerotic lesions in cardiovascular disease, to name a few. It is evident that humans have been and will continue to be exposed to PCBs due to continual inputs into the environment from landfills and incinerators [40]. PCB153 is a persistent non-dioxin-like polychlorinated biphenyl (NDL-PCB) abundantly present in the food-chain and the environment. NDL-PCBs are known to be less toxic then dioxin-like (DL) congeners, but may still cause deleterious public health effects because of their tendency to bioaccumulate to high concentrations due to their slow elimination from the human body. In adults, the estimated elimination half-life for PCB153 is 14.4 years [41].

The effect of environmental PCBs on Id proteins has not been investigated until now which makes the results reported in this study novel. The major findings from this study are: (*i*) PCB-induced ROS mediated a highly tube branched neovascular phenotype that also depended on ID3 and Pyk2; (*ii*) PCB153 treatment increased the size of endothelial spheroids under conditions typically used for clonal selection of stem cells; (*iii*) High ID3 protein expression correlated with a greater degree of malignancy and oxidative DNA damage marker 8-OHdG in blood vessels; (*iv*) PCB153 increased ID3 protein independent of AhR signaling which was due to protein stabilization; (*v*) PCB153 treatment increased both serine and tyrosine phosphorylation of endothelial ID3; (*vi*) Pyk2 kinase phosphorylated ID3 *in vitro*; and (*vii*) ID3 overexpression increased cell survival of human microvascular endothelial cell line hCMEC/D3. Together, our results present evidence in the support of PCB-induced ROS-mediated Pyk2 signaling leading to the activation of ID3 which is presumably necessary for regulating vascular EC survival and development of microvascular lesions. These observations are important because they provide a new paradigm by which PCBs may contribute to the growth of microvascular lesions.

The molecular mechanisms responsible for the development of microvascular lesions in response to exposure to PCBs are not fully understood. Many environmental contaminants exhibit their adverse effects via ROS mediated signaling. A considerable body of evidence supports an important role for ROS in promoting microvascular lesions, at least in part, through the stimulation of growth factor signaling [42]. The majority of PCBs in plasma are transported by albumin [43]. The albumin binding protein gp60, which is localized to caveolae on the surface of ECs [44], allows for interaction between PCBs in plasma and caveolae-associated membrane signaling proteins that may include Pyk2. Pyk2 represents a novel member of the focal adhesion kinase family that is activated by ROS in vascular cells [37]. In the present study, we have not identified the detailed mechanism by which the PCB-induced ROS upregulated ID3 levels. However, our data indicate that there are post-translational effects that increase protein stabilization which have functional consequences for neovascularization. Our findings did show that PCB-induced Pyk2 activation depends on ROS in hCMEC/D3 cells. Pyk2 activation results from its autophosphorylation on Tyr-402, which creates a SH2 binding domain. Since the SH2 domain recruits other proteins to Pyk2, these protein-protein interactions may be important for signaling to ID3. Although it still needs to be confirmed *in vivo*, our data show that Pyk2 kinase can phosphorylate ID3. Thus, Pyk2 mediated signaling to ID3 may be one possible pathway for PCB-induced neovascularization. It is not clear whether PCB153-induced phospho-ID3 leads to protein-protein interactions or nuclear translocation that may prevent its degradation; however, our findings demonstrated that ID3 is a target of post-translational modifications by PCB153 in microvascular ECs. Results from the cardiovascular tissue microarray provide further support that ID3 plays a role in vascular disease as ID3 expression correlated with both grade of malignancy and 8-OHdG in blood vessels. If a marker of oxidative DNA damage 8-OHdG is used as a surrogate for oxidative stress, then it is plausible that oxidative stress commonly found in malignant tissues may mediate the high ID3 levels via ROS signaling pathways. It is known that cells exposed chronically to toxic chemicals adapt to survive and develop resistance to cell death. ID3 is not only activated by PCB-induced ROS, but we observed that PCB153 provided a growth advantage. We showed that PCB153 treatment increased the size of endothelial spheroids that is most likely due to increased cell survival and is corroborated by a report of increased cell survival by chronic *in vitro* PCB153 (50–100 µM) 12 wk exposure [45]. It is becoming increasingly recognized that the pathogenesis of microvascular complications includes disordered proliferation of ECs. Our data showed that ID3 overexpression increased cell survival; hence PCB-induced ID3 may support survival of damaged ECs that contribute to the formation of a microvascular lesion. The increase in cell survival may explain the formation of reported hyper-proliferative, apoptosis-resistant, and monoclonal ECs found in microvascular lesions.

Epidemiological studies of human PCB exposure have shown that PCB153 had the highest serum levels among PCB congeners [46]. The endothelial phenotype changes in our study were observed at a 1 ng/ml dose of PCB153 which is within the reported range of PCB153 [0.60–1.63 ng/ml] found in human serum [47]. Thus, our findings are of significant public health importance because they showed that endothelial phenotype changes occurred *in vitro* by reported doses of PCBs found in human serum. The mechanistic implications of our findings on PCB153-induced ID3 may be with its connection to metabolic syndrome. Metabolic syndrome is the name for a group of risk factors often characterized by oxidative stress that raises one's risk for cardiovascular disease and other health problems such as diabetes in which microvascular lesions are found. PCBs have been associated with metabolic syndrome in epidemiological studies [48]. In the mouse model, PCB153 has been shown to produce significant metabolic changes when administered with a high fat diet that were consistent with worsened obesity and nonalcoholic fatty liver disease pathology [49]. ID3 may contribute to metabolic syndrome via its effects on obesity because animal models have already shown that ID3-induced visceral fat expansion in mice fed a high-fat diet [50]. It was not known why ID3 deficient mice showed higher energy expenditure and higher metabolic rate at rest, although previous studies have indicated that ID3 is regulated by glucose in pancreatic islet β-cells [51]; and in macrophage cells under chronic hyperglycemic conditions the closely related family member Id2 was reported to be glycosylated and protein levels stabilized which in turn had functional effects on metabolic genes [52]. A causal link of diabetes and microvascular complications is chronic hyperglycemia. More importantly, however, is that mitochondrial ROS produced by ECs in hyperglycemic conditions may share a pathway similar to environmental toxicant induced oxidative stress which converges on ID3. Since the findings from our study showed that PCB153-induced ROS modulated ID3 protein levels and post-translational modifications, our future investigations are focused on how exposure to environmental PCBs may form a risk factor for the development of metabolic syndrome and altering metabolism through ID3 regulation.

In summary, the major novel findings which emerged from this study are that PCB-induced ROS-mediated Pyk2 signaling leading to the activation of ID3 is presumably necessary for regulating vascular endothelial cell survival and development of microvascular lesions. A better understanding of how microvascular lesions depend on ID3 molecular targets may open up new avenues for prevention and treatment of vascular diseases in populations sensitive to environmental toxicant exposures.

## Supporting Information

File S1 Contains the following files: **Table S1**. Tyrosine kinase/phosphatase motifs found on ID3. Table shows results from a search with the PhosphoMotif Finder program. Search results revealed that ID3 had 8 tyrosine kinase/phosphatase motifs described in the literature. **Table S2**. Serine kinase/Phosphatase motifs found on ID3. Table shows results from a search with the PhosphoMotif Finder program. Search results revealed that ID3 had 38 serine kinase/phosphatase motifs described in the literature.
(DOCX)
