# Supplementary figures and images for: PCB153-Induced Overexpression of ID3 Contributes to the Development of Microvascular Lesions
Source: PLoS One. 2014 Aug 4;9(8):e104159. doi: 10.1371/journal.pone.0104159 (PMC4121297; doi:10.1371/journal.pone.0104159)

**Table S1. Tyrosine kinase / Phosphatase Motifs**


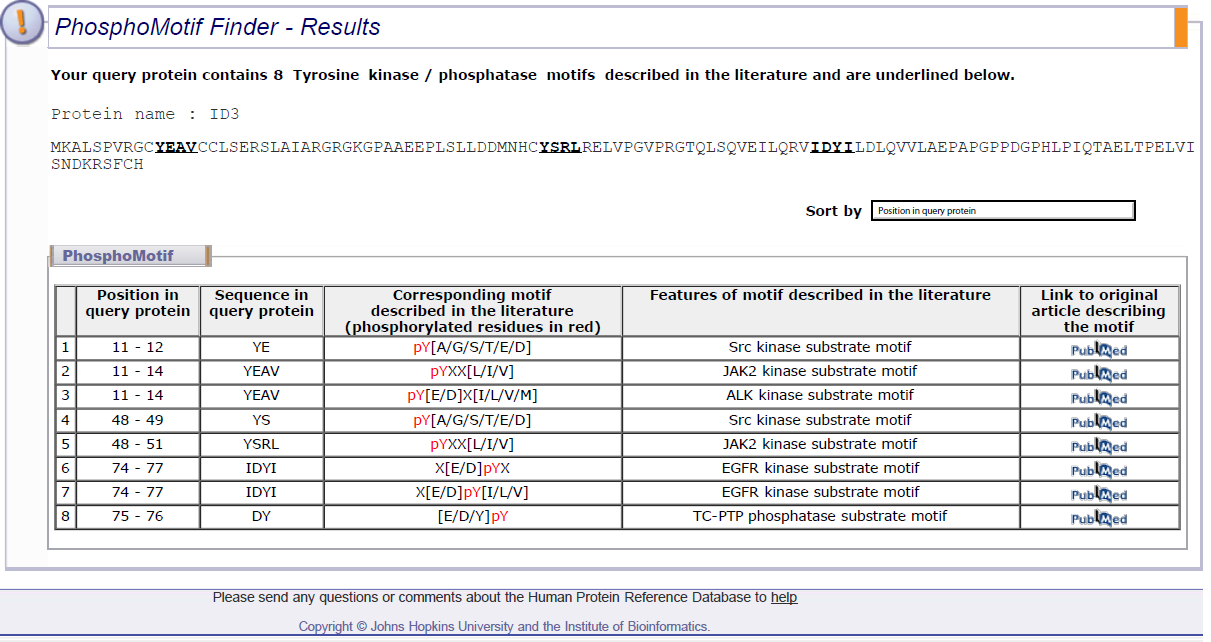


**Table S2. Serine kinase / Phosphatase Motifs**


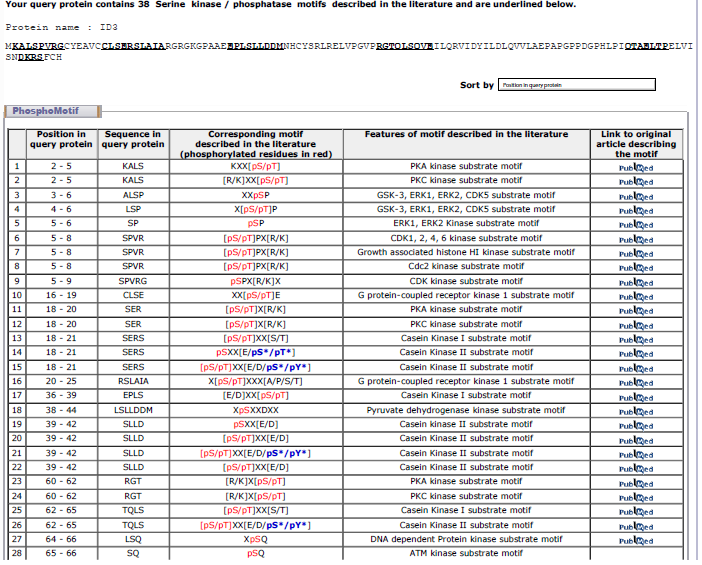

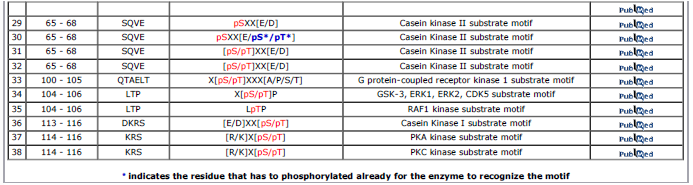

Supplement: File S1 — Contains the following files: Table S1. Tyrosine kinase/phosphatase motifs found on ID3. Table shows results from a search with the PhosphoMotif Finder program. Search results revealed that ID3 had 8 tyrosine kinase/phosphatase motifs described in the literature. Table S2. Serine kinase/Phosphatase motifs found on ID3. Table shows results from a search with the PhosphoMotif Finder program. Search results revealed that ID3 had 38 serine kinase/phosphatase motifs described in the literature. (DOCX) [file pone.0104159.s001.docx]
